# Supplementary material for: Electrical stimulation promoting the angiogenesis in diabetic rat perforator flap through attenuating oxidative stress-mediated inflammation and apoptosis
Source: PeerJ. 2024 Jan 31;12:e16856. doi: 10.7717/peerj.16856 (PMC10838069; doi:10.7717/peerj.16856)
Supplement: Supplemental Information 2 [file peerj-12-16856-s002.pdf]

Supplemental Figure S1-S3: original western blotting band

Electrical stimulation promoting the angiogenesis in diabetic rat perforator flap through attenuating oxidative stress-mediated inflammation and apoptosis

Authors: Cong Chen[1], Xiaolu Li[1], Yong Hu[1], Yuan Chen[2], Hongrui Wang[1], Xian Li[1✉], Xiucun Li[1✉]

1 Department of Hand and Foot Surgery, The Second Hospital of Shandong University, No.247, Beiyuan Street, Jinan, Shandong, 250033, China.

2 Institute of Medical Sciences, The Second Hospital of Shandong University, No.247, Beiyuan Street, Jinan, Shandong, 250033, China.

✉：These two authors contributed equally to this work, co-corresponding authors.

Corresponding Authors: Xian Li[1✉], Xiucun Li[1✉]

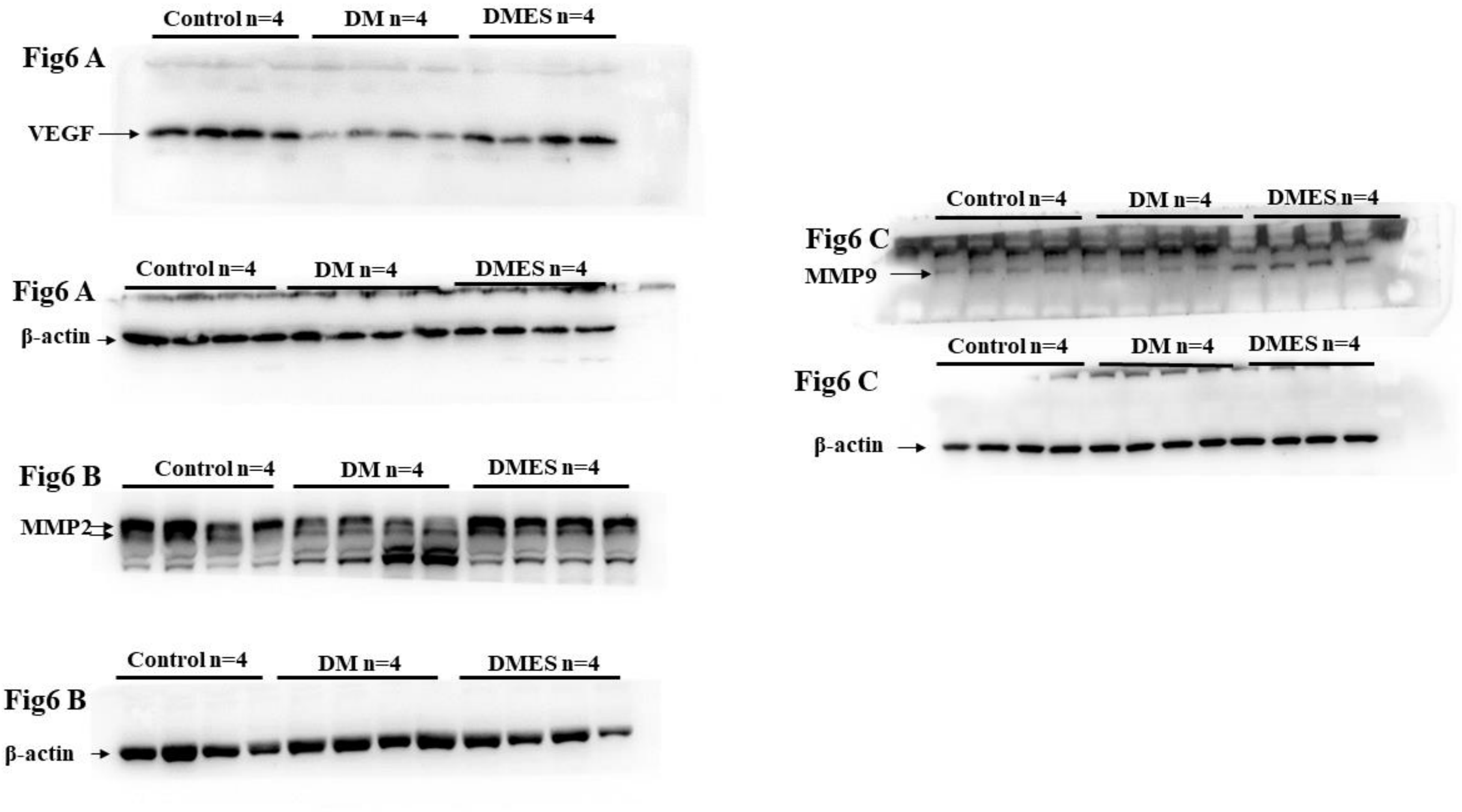

Supplemental Figure S1. The original western blotting images of the Figure 6 (Western blotting showing the expression and quantification of the optical density of VEGF (A), MMP2 (B), MMP9 (C) in the control, DM and DMES group).

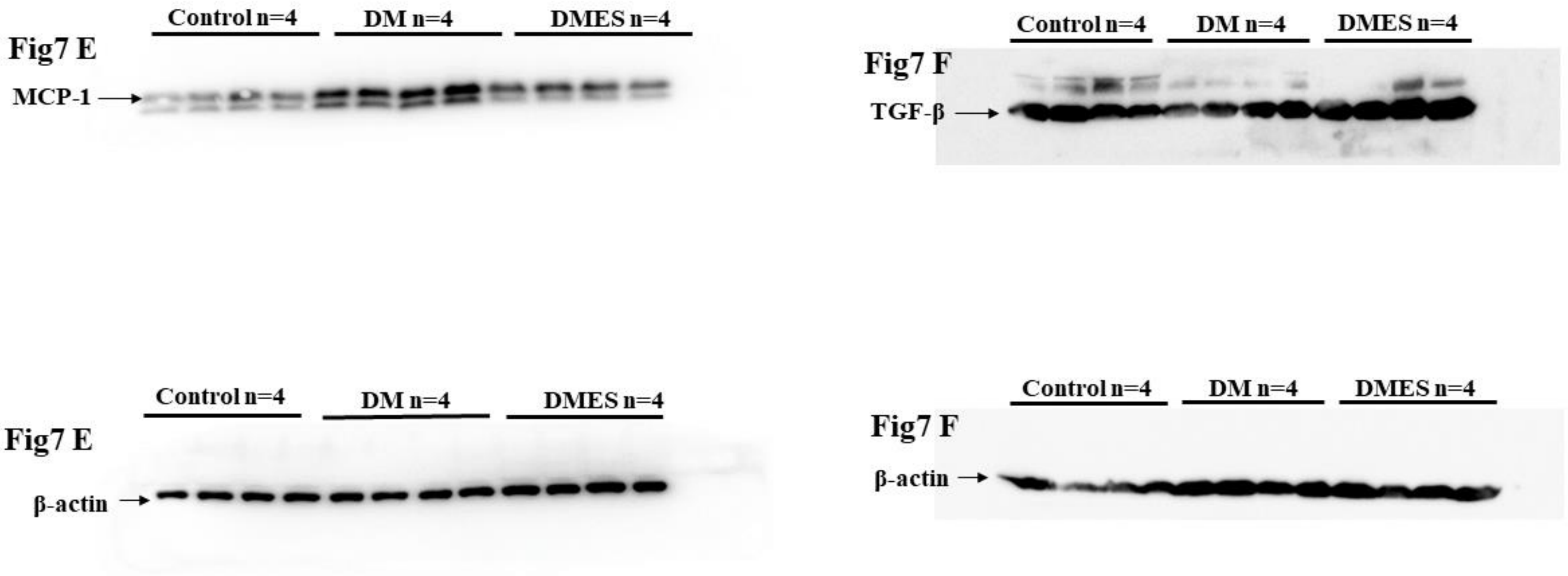

Supplemental Figure S2. The original western blotting images of the Figure 7E and F.

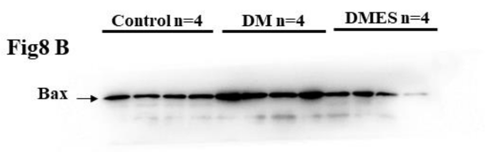

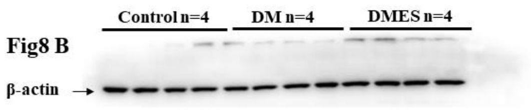

Supplemental Figure S3. The original western blotting images of the Figure 8B.
